# Genotyping-by-Sequencing Enhances Genetic Diversity Analysis of Crested Wheatgrass [*Agropyron cristatum* (L.) Gaertn.]

**DOI:** 10.3390/ijms19092587

**Published:** 2018-08-31

**Authors:** Kiran Baral, Bruce Coulman, Bill Biligetu, Yong-Bi Fu

**Affiliations:** 1 Department of Plant Sciences, University of Saskatchewan, 51 Campus Drive, Saskatoon, SK S7N 5A8, Canada; kiran.baral@usask.ca (K.B.); bruce.coulman@usask.ca (B.C.); 2 Plant Gene Resources of Canada, Saskatoon Research and Development Centre, Agriculture and Agri-Food Canada, 107 Science Place, Saskatoon, SK S7N 0X2, Canada

**Keywords:** genotyping-by-sequencing, *Agropyron*, genetic diversity, genetic structure, SNP

## Abstract

Molecular characterization of unsequenced plant species with complex genomes is now possible by genotyping-by-sequencing (GBS) using recent next generation sequencing technologies. This study represents the first use of GBS application to sample genome-wide variants of crested wheatgrass [*Agropyron cristatum* (L.) Gaertn.] and assess the genetic diversity present in 192 genotypes from 12 tetraploid lines. Bioinformatic analysis identified 45,507 single nucleotide polymorphism (SNP) markers in this outcrossing grass species. The model-based Bayesian analysis revealed four major clusters of the samples assayed. The diversity analysis revealed 15.8% of SNP variation residing among the 12 lines, and 12.1% SNP variation present among four genetic clusters identified by the Bayesian analysis. The principal coordinates analysis and dendrogram were able to distinguish four lines of Asian origin from Canadian cultivars and breeding lines. These results serve as a valuable resource for understanding genetic variability, and will aid in the genetic improvement of this outcrossing polyploid grass species for forage production. These findings illustrate the potential of GBS application in the characterization of non-model polyploid plants with complex genomes.

## 1. Introduction

Genotyping-by-sequencing (GBS) is a powerful genomic approach for identification of genetic variation on a genome-wide scale for genetic diversity analysis of non-model plants [1,2,3]. This approach produces high-density, low-cost genotypic information without the requirement for a reference genome sequence [4]. The detailed GBS approach in plant diversity analysis is described in Peterson et al. [3]. In brief, the GBS analysis involves five major steps: (1) genome complexity reduction with restriction enzyme; (2) barcoding the seared genomic DNAs with indexed adaptors; (3) high-throughput sequencing of barcoded DNA fragments; (4) identification of genetic variants through a bioinformatics analysis of de-multiplexed reads; and (5) a genetic diversity analysis of sequenced samples based on sample-by-variant matrix. The GBS application, despite being a powerful approach, has certain limitations, including many missing data points, uneven genome coverage, complex bioinformatics, and issues related to polyploidy [5,6,7,8]. To overcome these limitations, a GBS-based pipeline, called Haplotag, was developed by Tinker et al. [9], which can generate tag-level haplotype and single nucleotide polymorphism (SNP) data for polyploid organisms. This approach has been successfully applied in the study of diploid and polyploid genomes in oat (*Avena sativa*) [10,11,12] and genetic diversity analysis of northern wheatgrass (*Elymus lanceolatus* ssp. *Lanceolatus*) [13].

Crested wheatgrass [CWG; *Agropyron cristatum* (L.) Gaertn.] is one of the perennial species of the genus *Agropyron* that comprises 10–15 species in a polyploid series of diploid (2n = 2x = 14), tetraploid (2n = 4x = 28) and hexaploid (2n = 6x = 42) forms with the P genome [14,15]. *Agropyron* species are native to temperate-frigid grassland and sandy soils of Eurasia [14,16,17], and were first introduced to Canada in 1911 [16]. CWG is the most important commercial species of the crested wheatgrass complex in Canadian grasslands [18]. It is characterized by an extensive root system, making it drought tolerant and winter hardy. CWG is considered an important pasture grass for early spring grazing, providing highly palatable and nutritious forage [19]. This species is easy to establish, has strong competitive ability, tolerates insect predation, provides high forage yield, and can be managed for multiple harvests in a season [16,19,20]. It performs well on marginal lands and semi-desert environments to moist moderately saline soils [19,20]. Due to these features, this species can be used for land reclamation of abandoned croplands, burnt and degraded areas, as well as in erosion control [21]. It has persisted as a high yielding species compared to native forage species, even in 20- to 40-year-old pastures, despite heavy grazing and trampling [19,22]. In addition, CWG is also known to possess traits of interest, including disease resistance, tolerance to abiotic stress, and high yield, which have been utilized in wheat and barley breeding [23,24,25,26,27]. The palatability and nutrient content of CWG declines after anthesis, and it becomes less desirable for summer grazing [19]. Thus, a goal of present CWG breeding programs is to develop later maturing cultivars that would maintain nutritive value into the summer grazing season. Development of high forage-quality, late-maturing CWG cultivars is limited by the relatively long varietal development process, few studies to assess genetic variability of the germplasm, and lack of an effective marker system for marker-assisted and/or genomic selection/breeding. Recent RNA-seq studies in CWG have identified flowering time related genes and flowering related differentially expressed genes [28,29]. This emphasizes the need for genetic diversity studies of CWG for the management and utilization of proper genetic resources in a breeding program as exogamous perennial forage species are often morphologically comparable, though they are genetically highly heterogeneous and heterozygous [30,31]. An adequate level of genetic diversity is crucial for both germplasm adaptation and the long-term sustainability of plant communities [32].

Attempts have been made to assess genetic variability within and among the genus *Agropyron* using molecular markers like amplified fragment length polymorphism (AFLP) [18] and simple sequence repeat (SSR) markers [31,33,34]. The revealed variabilities have allowed for better understanding of the extent of diversity present in the genus. However, these marker systems are unable to provide high resolution of genetic diversity and population structure information to understand the ancestry and microevolution of the populations. Research is needed to assess molecular characteristics of CWG for plant breeding. The molecular characterization is now more feasible than before with the advanced sequencing technology and reduced cost to acquire informative markers such as SNPs in non-model polyploid CWG plants. Recent GBS studies in polyploid plants [10,13] demonstrate the likelihood that GBS will unveil genetic variability on a genome-wide scale in CWG plants, and characterize CWG germplasm for breeding and genetic research.

This study was conducted with the objective to apply GBS in combination with the Universal Network Enabled Analysis Kit (UNEAK) [35] and the Haplotag pipelines to (1) identify genome-wide SNP markers; (2) assess the genetic diversity present in 12 lines of *A. cristatum*; and (3) assess whether the GBS application is useful in the genetic diversity analysis of complex polyploid plants.

## 2. Results

### 2.1. SNP Discovery and Characterization 

**Figure 1:**
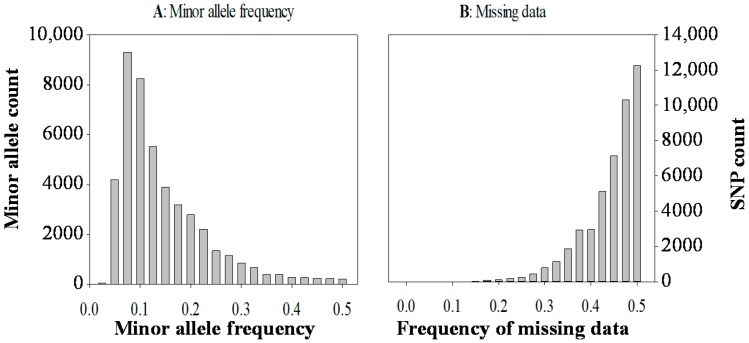
The minor allele frequency distribution (**A**) and the frequency of missing data (**B**) for 45,507 SNP markers in 192 genotypes of 12 crested wheatgrass lines.

The Miseq run of 192 genotypes from 12 CWG lines (Table 1) generated approximately 87.8 million raw forward (R1) sequence reads of 250 bp. The number of raw forward sequence reads per sample ranged from 190,606 to 775,160 with an average of 457,279. Combined UNEAK and Haplotag analysis at the 20%, 30%, 40%, and 50% level of missing data generated 227; 1,884; 10,738; and 45,507 SNPs, respectively across the 192 genotypes. In addition, this analysis also generated many metagenomic files associated with the SNP discovery, which are described and accessible in the online Supplementary Materials. The distribution of the minor allele frequency in 45,507 SNPs’ data ranged from 0.025 to 0.5, and exhibited a steady decline of minor alleles with increased occurrence of frequencies from 0.075 to 0.5 (Figure 1A). Likewise, there were more SNPs at the higher percentages of missing data (Figure 1B).

### 2.2. Genetic Structure and Relationship

**Figure 2:**
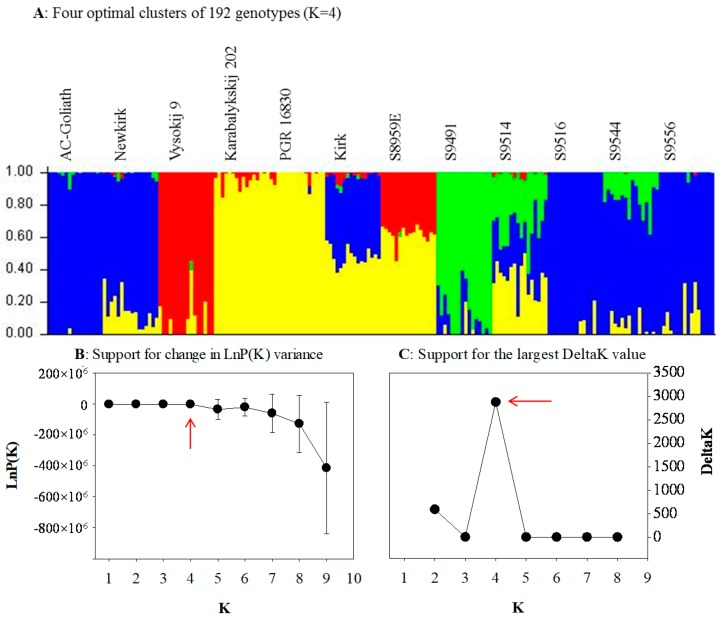
Four genetic clusters of 192 genotypes of the 12 crested wheatgrass lines inferred by STRUCTURE based on 45,507 SNP markers. (**A**) The mixture coefficients of 192 genotypes with K = 4, presented in the original order of genotypes from 12 lines (see Table 1 for line label); (**B**) support from the LnP(K) estimation; (**C**) support from the estimation of the largest value of the delta K = mean (|Ln”(K)|)/sd (LnP(K)).

**Figure 3:**
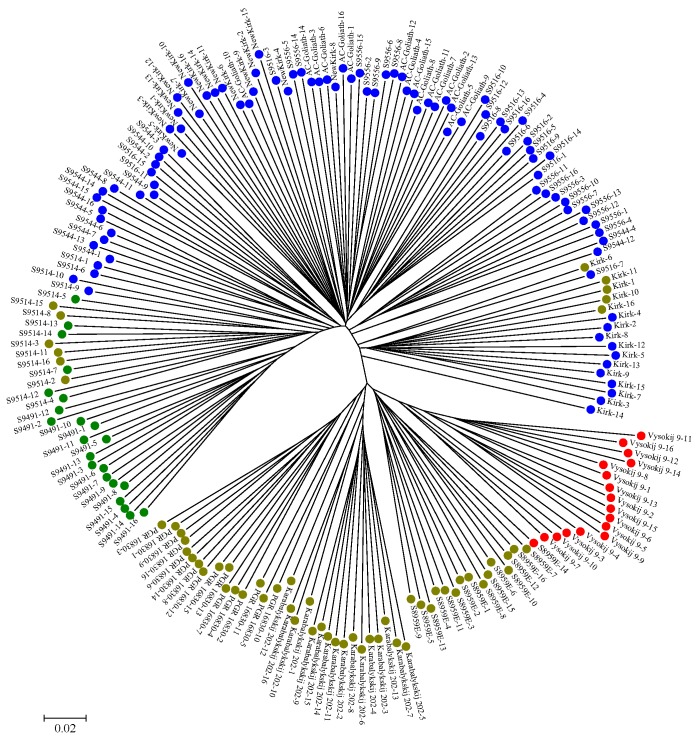
Genetic relationship of 192 genotypes of the 12 crested wheatgrass lines as revealed by neighbor-joining clustering with the 45,507 SNP markers. Each genotype is numbered after its line label. Each node for a genotype is represented with colored circle followed by genotype name. Red, green, blue, and yellow represent plants in Clusters 1, 2, 3, and 4, inferred from the STRUCTURE analysis (Figure 2A), respectively.

The genetic structure estimated for 192 genotypes from 12 CWG lines without consideration of prior population information in the STRUCTURE [36] analysis revealed four optimal clusters (Figure 2A) with strong support from change in LnP(K) variance (Figure 2B) and the largest delta K value (Figure 2C). Cluster 1 (red in color) consisted of 17 genotypes (16 from Vysokij 9 and one from S8959E). Cluster 2 (green in color) had 22 genotypes (16 from S9491 and 6 from S9514). Cluster 3 (blue in color) was the largest cluster, with 95 genotypes from seven lines. Cluster 4 (yellow in color), with 58 genotypes from five lines, was the second largest cluster. The neighbor-joining (NJ) tree was in agreement with clusters obtained from the STRUCTURE analysis (Figure 3). However, there existed some discrepancies, as some members of cluster 4 (yellow in color) were spread into cluster 2 (green in color) and cluster 3 (blue in color).

**Figure 4:**
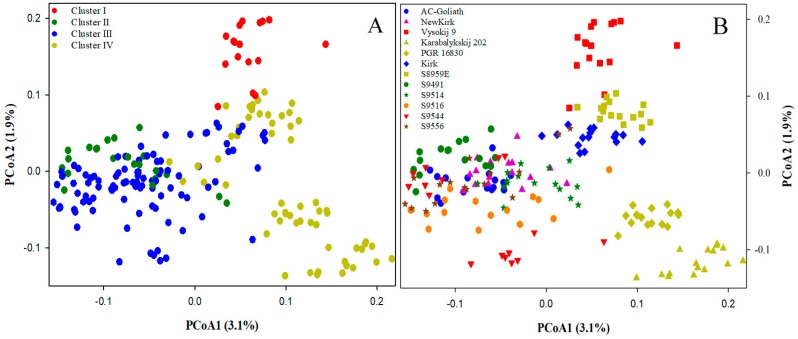
Genetic relationship of 192 genotypes of the 12 crested wheatgrass lines as revealed by principal coordinates analysis (PCoA) with the 45,507 SNP markers. Two panels are identical, but in the left panel (**A)** each genotype is labelled with colored circles representing the clusters obtained from the STRUCTURE analysis, while the right panel (**B)** labels genotypes for 12 lines.

The principal coordinates analysis (PCoA) revealed that the genetic relationship of 192 genotypes (Figure 4A) was not in accordance to the Bayesian inferences from the STRUCTURE analysis. The clusters II, III, and IV identified by the Bayesian inferences appeared to overlap and became undistinguishable with PCoA. However, the PCoA plot was able to distinguish four lines Karabalykskij 202 (from Kazakhstan), PGR 16,830 (from Kazakhstan), Vysokij 9 (from Russia) and S8,959E (selected from Vysokij 9) from the rest of the lines (Figure 4B). We also observed lines S9,516, S9,544 and S9,556 from cluster 3 (blue in color from the model-based Bayesian analysis) were more dispersed than other breeding lines and cultivars, likely indicating the larger genetic diversity present in those breeding lines (Figure 4B).

### 2.3. Genetic Differentiation

**Figure 5:**
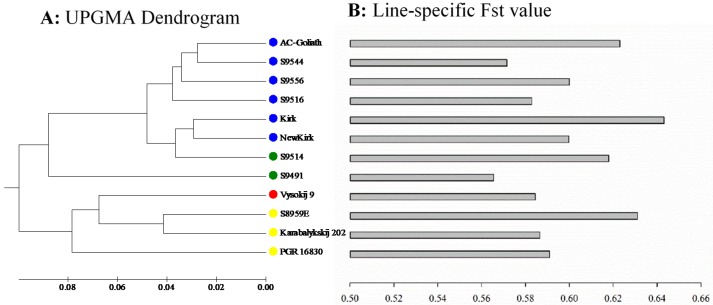
Genetic diversity and genetic relationships of the 12 crested wheatgrass lines. Left panel (**A**) shows their genetic relationship in the unweighted pair group method with arithmetic mean (UPGMA) dendrogram based on the Phi statistics obtained from the AMOVA. The right panel (**B**) displays the line-specific F_st_ values for the 12 lines.

The analysis of molecular variance (AMOVA) revealed that most of the SNP variations were present within the lines (84.2%), while much smaller variations reside among lines (15.8%) or among the four Bayesian clusters (12.07%) (Table 2). Line-specific F_st_ was also estimated from AMOVA for each line as the weighted variation among individual plants within a line to observe the extent of inbreeding. They were obtained in the range of 0.56 (in line S9491) to 0.64 (in the cultivar Kirk) with mean of 0.60 (Figure 5B). The pairwise genetic distance among the 12 lines ranged from 0.055 (between AC-Goliath and S9544) to 0.32 (between Karabalykskij 202 and S9491) with an average distance of 0.15.

The dendrogram based on AMOVA showed the grouping of the 12 CWG lines into three genetically distinct clusters at the Phi statistic of 0.08 or more (Figure 5A). The dendrogram grouped the lines from Kazakhstan and Russia in one distinct cluster. The second distinct cluster consisted of the single line S9491. The largest of all is the third cluster, with seven lines consisting of cultivars and breeding lines from Canada.

### 2.4. Effects of Missing Data on Diversity Analysis

**Figure 6:**
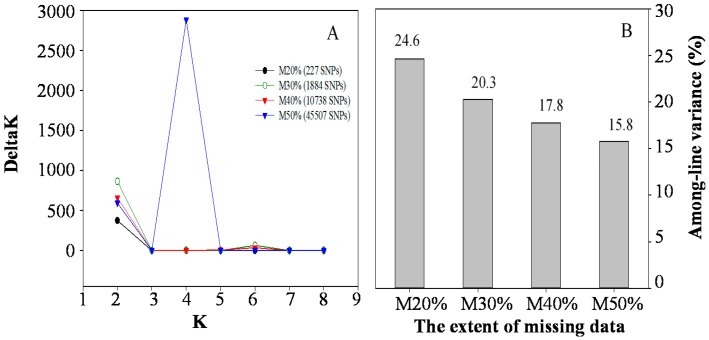
The impact of missing SNP data on the inferences of STRUCTURE and AMOVA analysis. The left panel (**A**) shows the four optimal clusters obtained from the STRUCTURE analyses at the missing level of M20% and M50%, and six clusters at M30% and M40%. The right panel (**B**) shows the SNP variances, ranging from 24.6 to 15.78%, inferred from AMOVA analyses residing among 12 lines at the increasing level of missing values from M20% to M50%, respectively.

The optimal numbers of genetic clusters inferred from STRUCTURE analyses with respect to the extent of missing data from M20%, M30%, M40%, and M50% datasets provided 4, 6, 6, and 4 optimal clusters, respectively (Figure 6A). Comparing the proportions of SNP variance residing among the 12 lines inferred from the AMOVA analysis showed 24.6%, 20.3%, 17.8%, and 15.8% for M20%, M30%, M40%, and M50%, respectively (Figure 6B).

## 3. Discussion

This study utilized the gd-GBS application, in combination with Haplotag pipeline, for the first time in CWG, to generate a data matrix of 192 genotypes × 45,507 SNP markers, and captured genome-wide genetic variants to evaluate the genetic diversity present in tetraploid CWG. The diversity analysis revealed 15.8% of SNP variation residing among the 12 lines and the model-based Bayesian analysis identified four major clusters of the assayed samples. These research outputs are not only useful for understanding the genetic diversity of CWG and for its breeding, but also are encouraging for molecular characterization of non-model polyploid plants.

The revealed patterns of genetic diversity are interesting. First, the model-based Bayesian approach in the STRUCTURE identified four major clusters of the assayed genotypes, while the distance-based approaches like PCoA and UPGMA identified three major clusters; however, the neighbor-joining analysis was in accordance with the result from STRUCTURE analysis. Following the pedigree of the assayed genotypes (Table S1), we could infer that the model-based Bayesian analysis and neighbor-joining analysis were able to genetically infer population substructure—an outcome of probable processes such as genetic drift, migration, mutation, and selection—more distinctly than distance-based approaches. Results also showed most of the genotypes grouped together within their lines, revealing that different lines were distinct. The STRUCTURE analysis (Figure 2A), neighbor-joining analysis (Figure 3), PCoA (Figure 4B), and UPGMA dendrogram (Figure 5A) revealed the genetic distinctness of lines Karabalykskij 202, PGR 16830, S8959E, and Vysokij 9. S8959E is a breeding line in the Saskatoon program, but it is a selection from Russian genebank line Vysokij 9. Although it has been recurrently selected for vigorous growth and plant type, it has not been interpollinated with any other lines, explaining its distinctness from other Canadian cultivars/breeding lines. However, STRUCTURE revealed all genotypes, except one (S8959E-14; Figure 2A) from line S8959E, showing high affinity with the line from Kazakhstan. This is also supported by UPGMA clustering (Figure 5A), while neighbor-joining analysis revealed the relatedness of lines from Russia. These findings will serve as valuable information for the genetic improvement of CWG for forage production.

Our analysis showed high within-line genetic variation (Table 2) of assayed CWG lines, which is in agreement with studies on highly outcrossing species [37]. Overall, our genetic diversity results are in accordance with diversity studies of CWG reported by Mellish et al. [18] using AFLP markers and Che et al. [31] and Che et al. [33,34] using SSR markers. The somewhat higher among population variation (15.8%) observed in the present study may partly be due to narrower genetic base of eight of the breeding lines/cultivars relative to the three genebank lines and one line of Russian origin (S8959E). Most of the Canadian cultivars and breeding lines shared one or more common parents in their genetic background (Table S1), and they have gone through many cycles of recurrent selection for vigor and yield. Thus, there has probably been a slight reduction in heterozygosity as indicated by the generally higher inbreeding coefficients (Figure 5B). The distinctness of the lines S8959E, Vysokij 9, Karabalykskij 202, and PGR 16830 can be attributed to their Asian origin and absence of interpollination with Canadian cultivars/lines and selection under Canadian conditions, except for the recurrent selection of line S8959E, mentioned above. Thus, the cultivars/breeding lines likely have reduced the within-line variation, while diverging more from the unselected Asian lines, explaining some increase of the among-line variation. Further research is needed on the utilization of the genetic variability of these lines with focus on morpho-physiological studies, adaptation, and their utilization in breeding programs. Likewise, the distinctness of the line S9491 in the UPGMA analysis (Figure 5A) is attributed to its synthesis from seven different lines/cultivars from breeding programs in Saskatoon and Logan, Utah, USA. The line S9514 was directly selected from S9491, which explains why these two lines clustered (green cluster) together in the STRUCTURE analysis (Figure 2) and neighbor-joining analysis (Figure 3). However, the Canadian cultivar “Kirk” developed partly from a plant introduction from a botanical garden in Finland (University of Turku) in 1968 showed shared pedigree with some or all of the Kazakhstan lines based on model-based Bayesian clustering (Figure 2A) and neighbor-joining analysis (Figure 3). While the origin of the plant introduction from the University of Turku remains unknown, it can be reasoned that this original introduction may have common genetic background with some of the Kazakhstan lines based on Bayesian clustering.

It was observed that the extent of reduction in heterozygosity, as explained by F_st_, was more in cultivars than most of the breeding lines. Two cultivars “AC-Goliath” and “Kirk” had lower diversity as indicated by higher inbreeding coefficient (F_st_ values) (Figure 5B), perhaps because of being synthesized from the interpollination of fewer genotype than many of the breeding lines. Also, most of the breeding lines included cultivars “Kirk”, “AC-Goliath”, and other sources, in their pedigrees. The cultivar “Newkirk” was selected from progenies of crosses between “Kirk” and “AC-Goliath”. However, the inbreeding coefficient of “Newkirk” was lower than the parental cultivars, indicating a higher level of heterozygosity. The three breeding lines S9516, S9544, and S9556 showed high within-line genetic diversity according to greater dispersal of these lines on PCoA (Figure 4B), higher within line variation (92.2%) as explained by a separate AMOVA, and lower line-specific F_st_ (Figure 5B). This greater genetic diversity could be attributed to inclusion of diverse germplasm sources during their synthesis (Table S1). The high within-line variability suggests that there is sufficient genetic variation in all lines in this study to make progress from selection. Inclusion of germplasm from the Asian lines in the breeding program to interpollinate with Canadian cultivars/breeding lines will increase diversity.

Our gd-GBS application has identified thousands of genome-wide SNP markers to assess the extent of genetic diversity in the non-model polyploid CWG with no prior genomic information. These results demonstrated the technical feasibility and effectiveness of GBS to sample genome-wide genetic variability in other perennial grass species with complex genomes. High resolution plant genetic diversity analysis, with 45,000 SNP markers spread over a genome, is more informative than with relatively few markers, like AFLP and SSR used in previous studies [1,12,18,38,39,40]. Also, the experimental cost for sampling genome-wide variants in this study was roughly $12,000, suggesting the feasibility of a wider application of GBS to characterize other perennial polyploid grass species. The results of the present study, along with those published in northern wheatgrass and wild oat [12,13], demonstrate the utility of GBS in molecular characterization of non-model plants with complex ploidy and genetic structures.

## 4. Materials and Methods

### 4.1. Plant Materials 

The study material comprised 12 tetraploid CWG lines consisting of six breeding lines, three cultivars, and three genebank accessions (Table 1). These accessions were acquired from USDA-ARS plant germplasm system, Plant Gene Resources of Canada (PGRC), and the joint forage breeding program of the University of Saskatchewan and Agriculture and Agri-Food Canada (AAFC). For ease of interpretation, all the acquired material will be referred to as lines, rather than accessions, in this study. Seeds of each line were grown for six weeks in the greenhouse at the Saskatoon Research and Development Centre, AAFC, under the following growth conditions: 16 h photoperiod at 22 °C and 8 h dark at 16 °C. Young leaf tissues were collected from 16 randomly selected plants for each of the lines and stored at −80 °C prior to DNA extraction. A total of 192 genotypes from the 12 tetraploid lines, listed in Table 1, were used for bioinformatics and genetic diversity analyses.

### 4.2. Genotyping-by-Sequencing

For each of the 192 genotypes, DNA was extracted from 0.1 g finely ground tissue following the protocols of NucleoSpin^®^ Plant II Kit (Macherey-Nagel, Bethlehem, PA, USA), and was eluted in a 1.5 mL Eppendorf tube with Elution Buffer. NanoDrop 8000 (Thermo Fisher Scientific, Waltham, MT, USA) was used to measure the quality of the DNA by comparing the 260 and 280 nm absorptions. DNA samples were further quantified through the Quant-iTTM PicoGreen® dsDNA assay kit (Invitrogen, Carisbad, CA, USA) and diluted to 60 ng/μL with 1× TE buffer prior to sequencing analysis.

A genetic diversity-focused GBS (gd-GBS) protocol by Peterson et al. [3] was used for the preparation of multiplexed GBS libraries. In brief, for each library, 200 ng purified genomic DNA was first digested with the restriction enzyme combination *Pst*I and *Msp*I (New England Biolabs, Whitby, ON, Canada). Ligation of customized adapters onto the 5′ and 3′ ends of the restriction fragments by T4 ligase was subsequently carried out. Then, the ligation fragments were purified by an AMPure XP kit (Beckman Coulter, Brea, CA, USA). Following the purification, Illumina TruSeq HT multiplexing primers were added through PCR amplification. The amplicon fragments were further quantified, concentrated, and pooled to form 4 subgroups of 12 samples each. The samples in the subgroups were pre-selected using a Pippin Prep instrument (Sage Science, Beverly, MA, USA) for an insert size range of 250–450 bp, before pooling the samples into a library. Each pooled library was diluted to 6 pM, and denatured with 5% of sequencing-ready Illumina PhiX Library Control (Illumina, San Diego, CA, USA) that can serve for calibration. Sequencing was completed using an Illumina MiSeq Instrument with paired-ends of 250 bp in length. MiSeq runs generated 384 FASTQ sequence files from 192 genotypes of 12 lines (one forward and one reverse for each of 192 genotypes). All the raw pair-end sequencing data in FASTQ format were deposited into the National Center for Biotechnology Information (NCBI) Sequence Read Archive (SRA) with accession number SRP115373 as part of the larger sequencing effort to enhance crested wheatgrass breeding [41]. The sequencing information for all 192 assayed samples is described in the online Supplementary Material, Section A.

### 4.3. Bioinformatics Analysis

Bioinformatic analysis began with sequence (FASTQ) data cleaning, using Trimmomatic version 0.36 [42] to remove any sequenced-through Illumina adapters, low quality sequence (sliding window of 10 bases, average Phred of 20), and fragments under 64 bases long.

As the UNEAK-GBS pipeline [35] only considers sequences of 64 bp (after barcode removal) with an intact 5-base *Pst*I residue (TGCAG) at the beginning, each FASTQ file of 250 bp was first split into three fragment sets with a custom Perl script *fastq184CutandCode-Pst.pl*. The first set comprised the first 64 bases with the *Pst*I residual restriction site, and the next two sets each with 59 base portions and an added 5-base *Pst*I residue. The script also provided an arbitrary barcode sequence (CATCAT) at the start of each sequence fragment, since the UNEAK pipeline expects to deconvolute barcoded sequence reads which are not already separated by sample. The three 70-base-long fragments formed, thereafter, were independent, as their relationship was not preserved. Each fragment set was recognized by the UNEAK-GBS pipeline [35], and was passed into UNEAK as an independent dataset.

Each fragment set (70 bases long) was analyzed with UNEAK and the Haplotag pipelines [9], resulting in the analysis of a total of 177 bases of genetic sequence. Online Supplementary Material, Section B, describes the procedures to run UNEAK. Two types of meta data files—a single mergedAll.txt (all tags observed more than 10 times) and a set of individual tagCount files (one per sample) needed for the Haplotag pipeline—were generated from the UNEAK run.

Haplotag was run with the parameters and filtering threshold settings described in the HTinput.txt file, and generated a matrix of samples by SNP loci (online Supplementary Material, Section B). A set of tag-level haplotypes (“HTgenos”) are first generated by Haplotag, followed by a set of SNP data derived from these haplotypes (“HTSNPgenos”). These two data types are technically redundant, so choosing one of them relies on the implementation and preference of software. In the present study, most (97.5%) haplotypes were found to contain only a single SNP; thus, we decided to analyze the SNP dataset for simplicity and compatibility with downstream analysis software.

The character by Taxa (CbyT) program supplied by N. Tinker was used to generate a filtered SNP file. In brief, Haplotag generated three separate “HTSNPGenos” files, which were merged before running CbyT. The “minimum presence” value in CbyT was set to 80%, 70%, 60%, and 50% for 20%, 30%, 40%, and 50% missing data, respectively. A SNP-by-sample matrix in the output files was used in further analyses. Additional descriptions of the SNP data matrix and the custom Perl and Shell scripts are available in the online Supplementary Material, Section A. Analyses from FASTQ file separation to SNP generation were conducted using Microsoft Windows 7 64-bit OS with an Intel (R) Xeon (R) CPU E5-2623 v3 @ 3.00 GHz (8 threads) and 32 GB RAM.

### 4.4. Genetic Diversity Analysis

The diversity analysis was based on 45,507 SNP markers, with 50% or less missing values in 192 genotypes from 12 CWG lines. Data analysis began with calculation of the minor allele frequency and the extent of missing SNP data with Microsoft Excel^®^. Thereafter, diversity analyses at the individual and line levels were carried out.

Three types of diversity analysis were performed at individual genotype level. First, genetic structure of 192 CWG genotypes was examined using a model-based Bayesian method implemented in the program STRUCTURE version 2.2.3 [36,43]. Linux server with 60 core parallel computing was used to run the STRUCTURE program, where each population subgroup (K = 1–9) was run 20 times, using an admixture model with 10,000 replicates each for burn-in and during the analysis. Based on (1) a plot of likelihood of these models, (2) the rate of change in the second derivative (∆K) between successive K values [44], and (3) the consistency of group configuration across 20 runs, the final population subgroups were determined. For a given population subgroup (K) with 20 runs, the run having the highest likelihood value was chosen to assign the posterior membership coefficients to each sample. These posterior membership coefficients were used to create a graphical bar plot. The size and formation of each optimal cluster with respect to population were evaluated. Second, a neighbor-joining (NJ) analysis of the 192 genotypes was conducted using MEGA version 7.0.14 [45] based on the dissimilarity matrix obtained from R routine AveDissR [46,47], and a radiation tree was displayed. Third, a PCoA of all 192 genotypes was also done using the R routine AveDissR [46,47] to assess genetic distinctness and redundancy, and to assess the genotype associations, plots of the first two resulting principal components were generated. For comparison, the resulting NJ trees and PCoA plots were individually labeled for the inferred structures.

Genetic variation present among the 12 lines was evaluated with AMOVA using Arlequin version 3.5 [48] on 45,507 markers. In addition, the pairwise genetic distances were computed and line-specific F_st_ values (inbreeding coefficient) for each line [49] were generated to infer the reduction in heterozygosity. To inspect the genetic variation among the clusters identified from the STRUCTURE analysis, additional AMOVA was performed. Unweighted pair group method, with arithmetic mean (UPGMA) dendrogram based on pairwise genetic distances among the 12 lines obtained from AMOVA, were generated using MEGA version 7.0.14 [45], to evaluate line differentiation and distinctness.

To estimate the influence of missing SNP data on the genetic diversity analysis, four datasets of 272; 1884; 10,738; and 45,507 SNPs representing 20%, 30%, 40%, and 50% of missing SNPs (M20%, M30%, M40%, and M50%) were attained for the 192 genotypes, respectively. For each dataset, the among-line variance from AMOVA and the optimal number of genetic clusters from STRUCTURE were obtained and compared among the four datasets of varying percentages of missing data.

## 5. Conclusions

With the application of GBS, it has been possible to generate 45,507 SNP markers for a diversity analysis of crested wheatgrass. The variation residing among these 12 lines of CWG was found to be 15.8%. Further analysis grouped the assayed samples into four genetic clusters, and revealed the genetic distinctness of two cultivars each from Kazakhstan and Russia, respectively. These results can enhance parental selection for increased genetic variation and improved offspring performance in crested wheatgrass breeding. The findings in this study can also aid in the application of GBS in the characterization of non-model plants with complex genomes.

## Figures and Tables

**Table 1:** List of the 12 crested wheatgrass (*A*. *cristatum*) lines used in the study.

Lines	CN Number ^a^	Alternative Identification ^a^	Origin	Type
Kirk	CN108662	PI 536010	Canada	Cultivar
AC-Goliath	CN108673		Canada	Cultivar
NewKirk		FOR552	Canada	Cultivar
Vysokij 9	CN30995	PI 370654	Siberia, Former Soviet Union, Omsk region	Genebank line
Karabalykskij 202	CN31068	PI 326204	Kazakhstan, Former Soviet Union, Kustanai region	Genebank line
PGR 16830	CN43478		Kazakhstan	Genebank line
S8959E		FOR917	Siberia/Canada	Breeding line
S9491		S9491	Canada	Breeding line
S9514		S9514	Canada	Breeding line
S9516		S9516	Canada	Breeding line
S9544		S9544	Canada	Breeding line
S9556		S9556	Canada	Breeding line

^a^ CN number is the line identification in Plant Gene Resources of Canada, Agriculture, and Agri-Food Canada (AAFC), while the alternative identifications, including FOR or S, are from the joint forage breeding program of the University of Saskatchewan and AAFC, and PI is from plant inventory book, National Germplasm Resources Laboratory, USA.

**Table 2:** Results of the analysis of molecular variance for two models of genetic structure (12 lines and four clusters from the STRUCTURE analysis) based on 45,507 SNP markers.

Model/Source of Variation	df	Sum of Squares	Variance Explained	Variance (%) ^a^
*12 lines*				
Among lines	11	101,048.8	246.0	15.8
Within lines	372	488,598.0	1313.4	84.2
*Four clusters from STRUCTURE*				
Among clusters	3	54,736.5	193.3	12.1
Within clusters	380	534,910.3	1407.7	87.9

^a^ These variances were statistically significant from zero at *P* < 0.0001.

## Supplementary Materials

Supplementary materials can be found at http://www.mdpi.com/1422-0067/19/9/2587/s1.
