# Supplementary material for: Genotyping-by-Sequencing Enhances Genetic Diversity Analysis of Crested Wheatgrass [Agropyron cristatum (L.) Gaertn.]
Source: Int J Mol Sci. 2018 Aug 31;19(9):2587. doi: 10.3390/ijms19092587 (PMC6163524; doi:10.3390/ijms19092587)
Supplement: Supplementary file 1 [file ijms-19-02587-s001.pdf]

International Journal of *Molecular Sciences*

MDPI

Supplementary Materials

Genotyping-by-sequencing enhances genetic diversity analysis of crested wheatgrass [Agropyron cristatum (L.) Gaertn.]

These supplementary materials are divided into the following two sections:

Section A: List of Supplementary Materials (three files and five zip folders) and they are available online (DOI://10.6084/m9.figshare.7001414)

A1. Table S1: List of 192 genotypes, sequencing information and pedigree of 12 lines (Excel file)
A2. CWG-CbyT-50-SNP.txt (Text file for SNP data at 50% missing level)
A3. Explanations for Haplotag output files (pdf file)
A4. Four zip folders for Haplotag output files
(mergedAll-ABC.zip; HTML-A.zip; HTML-B.zip HTML-C.zip)
A5. One zip folder of 13 files for all the custom shell and perl scripts and related files

Section B: Detailed procedure for analyzing FASTQ files using UNEAK and HAPLOTAG to generate tag-level SNP data

1. GBS data

A total of 192 *A. cristatum* genotypes from 12 lines (Table 1 and Table S1) were evaluated in this study. Total genomic DNAs were extracted and digested with *PstI* and *MspI* using the gd-GBS method (Peterson et al. 2014). MiSeq sequencing runs each with 48 samples, generated a total of 384 forward (R1) and reverse (R2) FASTQ sequence files of 192 genotypes. The FASTQ data was trimmed with Trimmomatic (Bodger et al. 2014) to remove any sequenced-through Illumina adapters, low quality sequence (sliding window of 10 bases, average Phred of 20), and fragments under 64 bases long. In the following analysis, we only examined the forward sequence (R1) reads.

2. Fragmenting FASTQ data

Each input sequence was fragmented into three parts: the first 64 bases containing the *Pst*I residual restriction site, and the next two 59 base portions, using the custom Perl script *fastq184CutandCode-Pst.pl*. The script added a six-base *Pst*I sequence to the beginning of the last two sequences followed by a pseudo bar code sequence (CATCAT) in front of each sequence fragments. The fragmented sequence were each 70 bases long and included a barcode and a restriction site that would be recognized by the UNEAK-GBS pipeline (Lu et al. 2013). The fragments had to meet the full-length requirement of each fragment to be processed. The relationship between the three

fragments was not preserved going into UNEAK and each fragment set was passed into UNEAK as an independent data set.

3. Run fragmented FASTQ data using UNEAK

UNEAK, available from https://tassel.bitbucket.io/TasselArchived.html (Accessed: 2018/02/27), (from the Tassel v 3.0 GBS pipeline (Glaubitz et al. 2013); Tassel v 3.0 UNEAK pipeline (Lu et al. 2012)) was executed with the following conditions:

- plugin -UFastqToTagCountPlugin to identify the *PstI* enzyme (-e);
- plugin -UMergeTaxaTagCountPlugin set to collect a maximum of 250-million tags per tagCount file (-m) and each tag requiring a minimum of 10 reads (-c);
- plugin -BinaryToTextPlugin to convert the mergedAll and individual sample tagCounts from the UNEAK binary format to text format.

The resulting *mergedAll.txt* and *individual tagCount* files were passed to the Haplotag software (Tinker et al. 2016).

4. Analyze fragmented FASTQ data using HAPLOTAG

Haplotag, available from http://haplotag.aowc.ca/ (Accessed: 2018/03/04), was run for each of the three sets of tagCount files with the following conditions in the HTinput.txt file:

- @DiploidSNPGenos, true, {show homozygotes as diploids e.g. AA}
- @Verbose, true, {set true for detailed model selection in HTML reports}
- @ThreePlus, false, {set true to limit HTML reporting to models with >2 haplotypes}
- @reportallpp, true, {report passports for cluster even if there is no model (default=false) }
- @MaxThreads, 99, {use 999 for maximum possible, 99 for Maximum minus 1}
- @MinTagCount, 10, {set high to inspect only deep-sequenced tags}
- @MaxBaseDif, 3, {Maximum number of base mismatches to join tags in a cluster}
- @MinPres, 0.02, {minimum minor allele frequency}
- @MaxPres, 0.99, {maximum major allele frequency}
- @MaxQ, 300000000, {maximum total tags to inspect - for low memory, testing etc.}
- @MaxS, 100000000, {maximum total tag clusters to inspect}
- @MaxTagsToTest, 9, {maximum tags in a cluster, clusters with more are ignored}
- @RSite, PstI-MspI, {restriction site HinfI, or ApeKI, or PstI-MspI}
- @ThreshGeno, 0.4, {Threshold for minimum complete genotypes (% of taxa) when selecting a model}
- @ThreshHet, 0.1, {Threshold heterozygote frequency}
- @ThreshMAHet, 0.4, {Max. het. freq. by allele., Excludes high-het rare allele., Set to 1 for bi-parentals}
- @ThreshTrihet, 0, {Threshold trizygote frequency (3 haplotypes in one taxon)}
- @ThreshMultiHet 0, {Threshold multizygote frequency (4 or more haplotypes in one taxon)}
- @HetRatio, 0.1, {threshold ratio of tag count for minor allele - below this ratio call a homozygote}

and the following program steps:

- !ReadTaxaIDFile, .\HTTaxa.txt
- !ClusterMergedAll, .\mergedAll.txt, Build HTclusters and HThaplos directly from UNEAK mergedall file
- !ReadClusters, .\output\HTClusters.txt, You need to read clusters after they are built.,
- !ReadHaplotypes, .\output\HTHaplos.txt
- !MakeTagByTaxa, .\tagCounts-txt\, Tagcounts from UNEAK,
- !IdentifyAlleles, build the models and report the genotypes and passports

This resulted in SNP calls contained in the 'HTSNPGenos files' for each of the three sets. The header row was removed from the second and third files. Subsequently, the three HTSNPGenos files were concatenated into a single set of SNP calls. The concatenated file was used in additional SNP filtering using the in-house Character by Taxa (CbyT) program provided by N. Tinker. CbyT was run with four different filtering levels for minimum presence: 80% (representing 20% missing data), 70% (representing 30% missing data) 60% (representing 40% missing data), and 50% (representing 50% missing data). The CbyT output file generated from the Haplotag HTSNPGeno file was used as the final SNP data file. Diploid SNP calls were converted to haploid format by replacing all homozygous diploid values with the haploid equivalent using the Linux sed command: i.e.

```
sed 's/AA/A/g' all-HTSNPGenos.txt | sed 's/GG/G/g' | sed 's/CC/C/g' | sed 's/TT/T/g'| sed 's/--/-/g'> all-HTSNPGenos-singles.txt
```

Sample CbyT.bat batch file for 20% missing data:

```
set SNPRAW=.\all-HTSNPGenos-singles.txt
set HAPRAW=.\all-HTGenos.txt

CbyT %SNPRAW% httaxa.txt null ALL-HTSNPGenos_SNP.txt 7 0 10 1 80
CbyT %HAPRAW% httaxa.txt null ALL-HTGenos_HAP.txt 6 0 10 1 80

pause
```

This example of the CbyT.bat file is set for:

- 7 = HTSNPGenos input data (@DiploidSNPGenos = false) or 6 = HTGenos input data
- 0 = diversity data,
- 10 = Max Het% (maximum heterozygosity as a percent),
- 1 = Min MAF % (minor allele frequency as a percent),
- 80 = Min Pres (minimum completeness score as a percent, i.e. the reverse of "missing data")

5. Supportive analysis

5.1 UNEAK key.txt files and Haplotag HTTaxa.txt and HTinput.txt files were prepared using a combination of Notepad++ and MS Excel.

5.2 Supportive Perl and shell scripts were specifically written and used to assist in the preparation of the various files required to run UNEAK and Haplotag and run from the Windows command (cmd) terminal or Cygwin (mintty 1.2-beta1 (x86_64-pc-cygwin; 2013) terminal:

- *fastq184CutandCode-Pst.pl* was used to prepare the input MiSeq fastq files for UNEAK,

- *tagCountTXTmaker.sh* was used to make the batch file to convert tagCount binary files to text files
- *tagCountTXTbatch.bat* converted the tagCount binary files to text files.

The above three text files and the UNEAK and Haplotag batch and info files are available as online supplementary information as described in section A.

5.3 UNEAK and Haplotag were run using Microsoft Windows 7 64-bit OS with an Intel (R) Xeon (R) CPU E5-2623 v3 @ 3.00 GHz (8 threads) and 32 GB RAM.

6. References

Bolger, A.M.; Lohse, M.; Usadel, B. Trimmomatic: A flexible trimmer for Illumina sequence data. *Bioinformatics* **2014**, *30*(15), 2114–2120, https://doi.org/10.1093/bioinformatics/btu170

Glaubitz, J.; Elshire, R.; Casstevens, T.; Harriman, J.; Buckler, E. TASSEL 3 Genotyping by Sequencing (GBS) pipeline documentation. **2013**, https://bytebucket.org/tasseladmin/tassel-5-source/wiki/docs/TasselPipelineGBS.pdf (Accessed: 2018/03/13)

Lu, F.; Glaubitz, J.; Harriman, J.; Casstevens, T.; Elshire, R. (2012). TASSEL 3.0 Universal Network Enabled Analysis Kit (UNEAK) pipeline documentation. *White Paper* **2012**, 1–12.

Lu, F.; Lipka, A.E.; Glaubitz, J.; Elshire, R.; Cherney, J.H.; Casler, M.D.; Buckler E.S.; Costich, D.E. Switchgrass Genomic Diversity, Ploidy, and Evolution: Novel Insights from a Network-Based SNP Discovery Protocol. *PLoS Genetics* **2013**, *9*(1), https://doi.org/10.1371/journal.pgen.1003215

Peterson, G.W.; Dong, Y.; Horbach, C.; Fu, Y.B. Genotyping-by-sequencing for plant genetic diversity analysis: A lab guide for SNP genotyping. *Diversity* **2014**, *6*(4), 665–680, https://doi.org/10.3390/d6040665

Tinker, N.A.; Bekele, W.A.; Hattori, J. Haplotag: Software for Haplotype-Based Genotyping-by-Sequencing Analysis. *G3: Genes|Genomes|Genetics* **2016**, *6*(4), 857–863, https://doi.org/10.1534/g3.115.024596

© 2018 by the authors. Submitted for possible open access publication under the terms and conditions of the Creative Commons Attribution (CC BY) license (http://creativecommons.org/licenses/by/4.0/).
